# A Treatise on Neuralgic Diseases, Dependent on Irritation of the Spinal Marrow

**Published:** 1830-01-01

**Authors:** 


### A TREATISE ON NEURALGIC DISEASES DEPENDENT UPON IRRITATION OF THE SPINAL MARROW AND GANGLIA OF THE SYMPATHETIC NERVE. By *T. Pridgin Teale*, &c. pp. 117. 1829.

THE influence of the spinal marrow upon a state of health or of disease is abundantly testified by the many and disastrous ailments which follow its derangement. But the precise nature of this influence has not been determined with sufficient certainty, the full extent of its results has not been fairly measured, and a thousand symptoms are every day ascribed to nervous irritation, without affixing to this ascription any practical import, or giving to the derangement which it is employed to designate any precise habitation. If pains be felt in the parietes of the chest, or integuments of the shoulders they are ascribed to rheumatism; if seated in the head they are denominated "*sick-headache*;" if we be oppressed with ennui it is because we are *bilious*; if tortured with convulsions then we are *nervous*. Cramp is explained when it is called colic; tremors when we are said to be irritable. The nervous system sympathises with some gastric disturbance if we complain of *tic*, and a few unmeaning vocables, as *nervous*, *bilious*, and *hysterical* are sufficient to supply us in all such affections with appropriate and abundant nomenclature.

But however unsatisfactory and ill-defined our knowledge of spinal maladies has hitherto been, the ganglionic system presents a subject of a still darker nature. The very origin of the sympathetic nerve was long unknown, if it be even yet universally agreed upon; and its functions and diseases are still placed by many in the *terra incognita* of the map of human knowledge. One considers it the grand conductor by which a thousand sympathies are created and preserved; another asserts that its ganglia may be torn, or dissected out without the animal betraying any consciousness of injury; and some go so far as to deny that it is a nerve at all. It was the opinion of Winslow that the sympathetic ganglia are reservoirs of nervous power;—of Johnstone, that they are the instruments by which the heart and intestines are endowed with involuntary agency;—of Bichat, that they are so many centres of organic life, *centres particulieres de la vie organique*, independent both of the brain and spinal marrow. These are only a meagre specimen of the many contradictory conjectures which have been hazarded upon the nature of a system, a part of which only Wrisberg called the *cerebrum abdominale;* and as to its pathology nothing was precisely known until within the last few years, when a more philosophical mode of studying such matters elicited more light, and when the valuable works of Soemmering, Scarpa, Philip, Hastings, and Lobstein enabled us to speak with more satisfaction upon the natural and morbid influence of the ganglionic system.

In the present little volume Mr. Teale has done much to rescue these interesting portions of the nervous system out of their obscurity; to give a precise meaning to terms which were as extensively employed as they were utterly unintelligible; to trace many wandering and anomalous symptoms to their proper source; to ascertain the character of that morbific cause on whose existence they depend; and to prescribe the treatment which is best adapted for their removal. By an attentive investigation of his subject he has certainly settled one most practical question—that disease of the spinal cord and sympathetic ganglia is often less strikingly evinced by symptoms exhibited in its immediate neighbourhood, than by such as may be discovered in distant parts; and by linking the seat with the symptoms of disease, he has given a clue to their treatment as well as to their etiology, which we have good reason to believe will frequently lead to their alleviation, when it fails to reach their cure.

Irritation of the Spinal Marrow.—In vindication of his view that an irritable state of the spinal marrow frequently occasions neuralgic affections in distant parts, the author furnishes nothing which can be called incontrovertible evidence; but the presumption which he establishes in its favour must be considered strong, where it proves not satisfactory. The frequent coincidence of tenderness in the spinal column with these neuralgic symptoms, the relief or aggravation of these symptoms as this tenderness diminished or increased, the obvious influence which treatment directed to the spine exerted upon the immediate seat of neuralgic disease, and the well ascertained pathological fact that some of the most marked effects of disease of the brain and spinal cord are discoverable, neither in the substance or vicinity of the disease, but in organs at a distance from the seat of action; —these are the leading considerations whereon the author rests his views, and it must be admitted that they are more than plausible. Dissection would certainly give more direct proof, but in the present stage of this inquiry it is not forthcoming, and until more extensive opportunities for investigation shall furnish autopsic light, we can only weigh with caution the evidence we possess and square our inferences with the extent of our information. If a person complain of darting pains in the intercostal spaces between the fourth and seventh ribs of the right side, if these spaces be tender under pressure, and if the intercostal muscles occupying them be occasionally affected with spasm, if a dull sense of heat and uneasiness be felt in the third and fourth dorsal vertebræ, if the pains of the chest appear to shoot from and to these vertebræ, and if on examining the spine this portion of it alone betray decided tenderness on pressure; if cupping, leeching, and blistering this portion of the spine be followed with marked alleviation of every symptom, and if with the removal of the spinal tenderness every indication of thoracic disturbance disappear, it is as certain as circumstantial evidence can make it, that there is some etiological relation between the tenderness of the spine and the pain in the chest, and that although we may not have the sanction of actual sight to fix the nature of this relationship, there can be as little practical as pathological hazard, in concluding that the seat of disease is principally, if not exclusively confined to the spinal column.

The same evidence which lights us to this conclusion will aid in discovering the nature of this disease. When the symptoms which attend it are compared with such as depend on acute inflammation of the spinal cord, they will be found to differ less in nature than degree. The introductory symptoms of both diseases are nearly, if not precisely the same, the sensibility of the skin is not destroyed, but impaired, the muscles are not palsied, but seized with feebleness and tremors. In the absence of better proof from the author, we refer to some of Dr. Abercrombie's cases, as given in his valuable work upon diseases of the brain. In his CXIth. case the patient complained of pain in different portions of the spine stretching round the abdomen, a very uneasy sense of tightness across the lower part of the chest, spasm of the abdominal and dorsal muscles, coldness and numbness along the sides of the chest, abdomen, and down the lower extremities, diminished power of motion and occasional hiccup. These symptoms gradually increasing the right arm became paralytic, his speech impaired, coma came on, and he died. Upon dissection the whole cord was found of a pale colour and in a state of complete ramollissement, a part of the medulla oblongata, cerebellum and brain was similarly diseased.

The tenderness of the spine at that part which the nervous symptoms would indicate as the seat of mischief, is strongly corroborative of its inflammatory nature, and the treatment, which is found to remove it with the greatest certainty, being purely antiphlogistic, inculcates the same inference. As the inflammation is subacute and seated in organs of great delicacy, dissection itself might often fail to give much additional force to these circumstantial evidences; for as the author justly says—

"Although the parts after death may not exhibit any traces of inflammation, we are not warranted in concluding that they have not recently been the seat of disease. When the conjunctiva has been intensely injected from inflammation, or the skin the seat of redness from erysipelas, how slight are the traces of inflammation after death! And if the conjunctiva and the skin may be intensely red from acute inflammation, and yet exhibit scarcely any traces of disease after death, it is more than probable that the spinal marrow should be equally destitute of the marks of disease. Indeed, it would even be a subject for surprise if any permanent changes in this structure had been effected, since the diseases in question are presumed only to consist in the lighter shades of inflammation, seldom attaining those violent degrees of intensity which are attended with obvious disorganization." 68.

The symptoms produced will vary both in nature and extent with the portion of spine affected. If the upper cervical portion be diseased neuralgia of the scalp is not uncommon, and the direction of the pain is generally determined by the position of the nerve. Sometimes the pain is dull, frequently it is acute, or it may occur in paroxysms. Occasionally some stiffness of neck accompanies the other symptoms, the voice may lose its natural tones, or speaking may be attended with difficulty.

A healthy youth was attacked (on the 10th of August, 1829,) with giddiness and pain of occiput, which frequently darted across the crown to the forehead. This pain, although generally dull, was often acute, and a feeling of weariness was complained of about the shoulders. These symptoms assumed the form of paroxysms, which were most frequent in the morning and continued about two hours; they had been gradually increasing for several weeks, and they were accompanied with tenderness of the third and fourth cervical vertebræ. Leeches to be applied to the tender part of the spine. 14th. Pains return at the usual time, but in a very mitigated form, the vertigo is less and the patient is much better in every respect; blister to neck. (18th.) Vertigo and pain quite gone, and no tenderness of spine was now elicited on pressure.

It may be thought that had the leeches and blister been applied in this case to the head, the relief might have proved as sudden and effectual. The following case will be a sufficient reply to this suspicion.

Mrs. B. a week after her confinement, complained very much in the afternoon with a dull aching pain in the occiput, which extended along the parietal bones to the temples, and transversely towards the cheeks along the lower jaw. She also complained of a violent pulsation and distressing sound in the head, which she compared to the "beating of hammers." These symptoms had somewhat annoyed her previously to her confinement, and leeches had been applied to the temples but with the effect of aggravating them during the two following days. On examining the spine very great tenderness of the two upper cervical vertebræ was detected, a circumstance that had been hitherto overlooked, leeches were applied, immediate relief was procured, and the paroxysms never afterwards returned.

When the inferior cervical division of the spinal marrow is the part diseased, pains are felt in the arms, shoulders and breasts. Sometimes they follow the course of the anterior thoracic branches of the brachial plexus, occasionally they are fixed in the neighbourhood of the shoulder-joint, and again they shoot along the cutaneous nerves. The mammæ not unfrequently become acutely sensible, and, if the affection have continued long, feel knotty and irregular; pricking, numbness and a sense of cold are often felt in the arms, the elbows are stiff, the muscles are affected with cramps and spasms, the wrists are weak, the hands tremble and the fingers are almost insensible to such objects as they may contain. Females of retired and sedentary habits are obnoxious to these neuralgic symptoms in the upper extremities, and their modes of life have been generally considered a sufficient explanation.

Mrs. B. aged 53, mother of a large family, had been much addicted to rheumatism for the greater part of her life, and on the 10th of December, 1827, made considerable complaint of a fixed pain in the neck and between the shoulders, of darting pains over the occiput, of aching pains over the entire arms, of prickling sensations of the hand and numbness of the fingers. Abdominal muscles occasionally painful, lower extremities free, no fever, no cough, nor dyspnœa. Her disease, having been considered rheumatism, had been treated with the ordinary remedies but without much benefit, and finding that the two lower cervical and six superior vertebræ of the back betrayed tenderness when pressed, leeches were applied and a blister was ordered for the next evening. The irritation occasioned by the blister was very great, and while it continued the original symptoms were more severe; but as it subsided they gradually disappeared, and on the 29th of December she was not only free from pain, but felt a degree of muscular energy in her arms which she had not enjoyed for several years.

The sixth case will remind our readers of Sir A. Cooper's description of the "irritable breast," and we cannot impress the importance of such cases upon them more forcibly than by observing, that not a few have been doomed in consequence of similar symptoms to severe and fatal operations, and that many are permitted to wither under the blighting suspicion that they are the victims of some malignant malady, which can only be appeased with the sacrifice of life. An unmarried lady, aged 30, consulted the author on the 28th of August, 1828, for a painful affection of the right breast, which had annoyed her for several years, particularly at the menstrual period, but which had increased within the last four weeks to a degree of alarming intensity. When examined it appeared enlarged and irregular, and the slightest touch produced acute suffering. "Sharp darting pains" often shot across the breast into the right arm, which was slightly tumefied and felt weak, a constant gnawing sensation in the shoulder, arm and breast was complained of, and when the tips of the fingers were suddenly touched pain darted up the arm to the neck and head, and down to the breast. Pressure over the fourth cervical and three upper dorsal vertebræ gave great uneasiness. Excepting occasional dyspeptic fits and an habitual torpidity of bowels the general health was tolerably good; eight leeches to the tender vertebræ and a purgative of salts and senna. (12th.) Bowels well opened, pain easier; blister spine. (30th.) Irritation of blister has occasioned some fever, and produced a bad night, pain unchanged; an effervescing draught every four hours. (Sept. 3d.) Irritation from blister has subsided, breast less swollen and bears pressure without pain, arm can be moved with greater ease. (10th.) Pains have returned; blister spine. (17th.) Breast of natural size, pains quite gone, arm can be moved with considerable facility. (Aug. 26th, 1829.) Breast continues well, and, with the exception of an occasional feeling of numbness and weakness in the right arm, she is quite well.

If the upper part of the dorsal spine be affected symptoms similar to those now described are produced, together with a fixed pain in some part of the intercostal muscles, or pleurodynia; when the lower half is tender an oppressive sense of tightness across the epigastre, soreness along the costal cartilages, or in the course of the diaphragm, and pains in the abdominal and lumbar muscles are the most distinguishing phenomena.

On the 1st of January, 1828, Mr. H. aged 40, who had been in an unhealthy state for several months, complained of a fixed pain in the intercostal spaces between the third and seventh ribs of the right side of the chest, which was increased by deep inspiration. Besides this fixed uneasiness there were acute pains which darted from the spine across the chest and towards the shoulders, and sometimes to the scalp. These pains recurred at short intervals through the day, and were less frequent in the night, tongue furred, appetite defective, occasional flatulence, frequent dry cough, considerable emaciation. Bowels regular, pulse natural. Having suffered for ten years from pain in the side he was considered to be labouring under phthisis; he had received much medical attention without improvement, and leeches and blisters had been applied in vain to the painful part. On examining the spine the third and fourth dorsal vertebræ betrayed very considerable tokens of morbid sensibility, and when the attention of the patient was drawn to this circumstance it occurred to him, that this part had often been the seat of uneasiness and unusual heat, and that the "darting pain in the chest appeared to strike to and from that part." From the first to the 25th of January dry cupping and leeching were twice employed, a blister was thrice and a sinapism once applied with decided relief; after the 25th a liniment containing oil of turpentine kept the integuments covering the tender vertebræ in a state of irritation, an effervescing draught was occasionally given to allay thirst, some rhubarb to keep the bowels open and sulphate of quinine to improve appetite and restore strength; and upon the 30th of February he considered himself perfectly recovered. If the lumbar or sacral part of the spinal column be affected the scrotum and neighbouring part often feel sore, the lower extremities are attacked with spasms, tremors and other morbid sensations, the knees totter and a sense of feebleness is complained of.

Mr. B. aged 20, complained of "pains across the body, weakness in the lower extremities, and soreness in the thighs" for the last three weeks. The abdominal pain is fixed, the soreness of the thighs descends along their interior over the knees to the broad surface of the tibiæ, his legs totter and he frequently feels a tendency to fall. Stomach in good health, and he seems free from any internal disease. In consequence of finding that the second and third lumbar vertebræ were tender ten leeches were applied, and a dose of oil was given. Five days afterwards the soreness and weakness of thighs and legs were removed, and the abdominal pain, which was much diminished, entirely disappeared on the application of a blister to the back.

An attentive perusal of the cases now extracted will enable the reader to form his own opinion as to the connexion between tenderness of the spine and such neuralgic symptoms. In some of them this connexion was obvious and easily traced by the character of the symptoms alone; in others it was strikingly presumptive from the nature and result of the treatment, and in all when the symptoms and treatment are viewed together, there can be little room for doubting that some morbid action within the spinal column was the principal source of every symptom. Mr. Teale calls this morbid action "irritation," and refers to the spinal marrow as its seat; but irritation is a vague and unmeaning epithet, which is better adapted to conceal ignorance than convey information, and the phenomena described in the above cases are indicative of inflammation. The acute and darting pains, the increase of heat, the occasional redness and even tumefaction, and the alleviation of all these symptoms by leeching, cupping, blistering and aperients, are strongly favourable to this opinion. In justice to the author, however, it must be noticed that while he has chosen to call this spinal disease *irritation* he affixes to the term, according to the French fashion, the idea of *subacute inflammation;* declining to use any stronger phrase until dissection had cast more light upon its pathology.

"This irritation, or subacute inflammatory state of the spinal marrow is not necessarily connected with any deformity of the spine, or disease in the vertebræ. It may co-exist with these as well as with any other diseases, but it so repeatedly occurs without them, that they cannot be regarded as dependant upon each other. Where, however, inflammation and ulceration of the vertebræ or intervertebral cartilages exist, it is probable they may predispose to, and, in some instances, act as an exciting cause of an inflammatory state of the nervous structures which they contain; for we not unfrequently find inflammatory affections of the vertebræ in conjunction with symptoms of irritation of the spinal marrow. But these two affections, although co-existing, bear no regular relation to each other; and, during the progress of the vertebral disease, the affection of the nervous structures is subject to great changes and fluctuations. The local remedies employed for arresting the disease in the bones, often alleviate the affection of the spinal marrow at the very commencement of the treatment, long before the vertebral disease is suspended; but as the neighbouring inflammation in the bones appears to predispose or excite the nervous mass which they contain, to disease, relapses of the nervous affections are repeatedly occurring during the whole course of the complaint.

"The affections of the spine, termed lateral curvature and excurvation, appear to have no necessary connexion with the disease which I have been describing; and the proportion of cases in which they are found united, is so small, that lateral curvature can scarcely be considered even as predisposing to this disease. The most extreme degrees of deformity frequently are observed without any affection of the nerves; and when lateral curvature does occasionally co-exist, local antiphlogistic treatment will often speedily remove the nervous symptoms, whilst the curvature remains unrelieved. Hence there is an impropriety in considering these nervous symptoms as a result of the deformity, and in explaining them upon the mechanical principle of pressure and stretching, to which the nerves are supposed to be subjected as they issue from the intervertebral foramina. If the pressure and stretching produced by the curvature, were the cause of the nervous symptoms, they ought to continue as long as the deformity remains.

"Symptoms of affection of the brain frequently occur in conjunction with these diseases of the spinal marrow. These however must be regarded as the result of extension of disease from one part to the other, most probably through the medium of the membranes. I shall however, purposely avoid touching upon these subjects, as it would be foreign to my present purpose to enter upon the discussion of cerebral neuralgiæ.

"*Treatment.*—When the different neuralgic symptoms which have been enumerated, can be traced to this morbid state of some portion of the spinal marrow, the treatment that ought to be pursued, is readily decided upon. Local depletion by leeches or cupping, and counter irritation by blisters to the affected portion of the spine, are the principal remedies. A great number of cases will frequently yield to the single application of any of these means. Some cases, which have even existed several months, I have seen perfectly relieved by the single application of a blister to the spine, although the local pains have been ineffectually treated by a variety of remedies, for a great length of time. A repetition of the local depletion and blistering is however often necessary after the lapse of a few days, and sometimes is required at intervals for a considerable length of time. In a few very obstinate cases issues or setons have been thought necessary; and where the disease has been very unyielding, a mild mercurial course has appeared beneficial.

"It is of course necessary that proper attention be paid to the regular functions of the bowels, and to the treatment, by appropriate means, of any other affection which may co-exist. It is needless, in this form of disease, to offer any directions respecting diet, as the judgment of every medical man will enable him to decide best on the general management of the case immediately under his notice.

"When my attention was first directed to this subject, I considered recumbency a necessary part of the treatment; it is, for a moderate length of time, undoubtedly beneficial, and frequently very much accelerates recovery; but subsequent observation has convinced me that it is by no means essential. I have seen several instances of the most severe forms of these complaints, occurring in the poorer classes of society, where continued recumbency was impracticable, which have, nevertheless, yielded without difficulty to the other means of the treatment, whilst the individuals were pursuing their laborious avocations.

"These observations, however, are not intended to apply to those cases in which there is actual disease of the vertebræ.

"When there exists a tendency to relapse, I have thought it advantageous to continue the use of some stimulating liniment to the spine for a few weeks after the other means of treatment have been discontinued. A liniment, consisting of one part of spirit of turpentine, and two of olive oil, is what has generally been employed." 21.

IRRITATION OF THE SYMPATHETIC GANGLIA. In the diseases which have been described the source of mischief lay in the spinal cord, and the effects of this mischief were extended to distant organs through the interposition of the spinal nerves; but those, upon which we are now entering, are said to originate in another source, and in place of arising from the spinal marrow are supposed to depend upon derangement of the ganglia of the sympathetic nerve. Upon first casting our eye upon the title page of this work, and seeing by it that the writer proposed to describe and treat diseases depending on the spinal marrow, and communicated by the cerebro-spinal nerves, apart from such as arose from the sympathetic ganglia, and were communicated by the nervous filaments derived from them, we admit that the proposal appeared to us little better than a vain refinement; but upon more mature deliberation we believe that the distinction herein established has a natural foundation. While this is granted, however, it must be evident that any distinction based upon such a difference must be purely nosological, and that it can have no influence upon the selection, or employment of our remedial plan.

"As the disease may be confined to one part of the spinal marrow, or may exist simultaneously in different portions, or may even pervade its whole extent, so the affection of the ganglia may be confined to one of these nervous masses, may exist in several which are contiguous, or in ganglia remote from each other; and, as there is reason to believe, the whole chain may occasionally be affected.

"The disease of the ganglia is seldom found, except in conjunction with that of the corresponding portion of the spinal marrow whereas the spinal marrow is often affected without the neighbouring ganglia being under the influence of disease. Thus we frequently find symptoms of disease in a portion of the spinal marrow without any evidence of its existence in the corresponding ganglia, frequently the symptoms of both combined, and occasionally, but rarely, symptoms referable to the ganglia, without the spinal marrow being implicated." 39.

The principal symptoms of an irritated state of the sympathetic ganglia are palpitations of the heart, asthmatic breathing, spasm of the stomach, neuralgic pains of the thoracic and abdominal viscera, and diseased secretions of the stomach, liver, and kidneys. Leucorrhœa is often attendant on these affections, but whether it be a coincidence or consequence the author finds it difficult to explain. Pyrosis he considers a neuralgic disease, and he is inclined to suspect that some forms of diabetes may partake of the same character.

The facts and arguments which were formerly employed to show that the disease of the spinal cord, which gives rise to neuralgia, was inflammatory, we now refer to as illustrative of the same point in the case of the sympathetic ganglia. Among two or three other cases Lobstein gives the history of a boy only ten years old, who died with symptoms of anxiety, oppression in the chest, and rattling at the pit of the stomach, in consequence of a repelled eruption, and in whom the trunk of the sympathetic nerve, the ninth and tenth thoracic ganglia, and two of the anastomosing branches were found upon dissection *profunde inflammata.* He has also seen the sympathetic destroyed *per ulcera et cariem*, but does not give the symptoms which occurred during life, and hypochondria, hysteria, melancholia, colica pictonum, angina pectoris, febris intermittens are only a tythe of the diseases which he describes as depending upon its derangement. Any of the sympathetic ganglia may be affected, but the middle and lower thoracic ganglia are those which are most frequently disordered, and the ganglia of the neck are affected next in frequency. As the stomach is chiefly supplied by the former and the heart by the latter, these organs, it may be supposed, should be the first affected; and hence do we find that the symptoms above enumerated are generally referrible to these viscera. So far, therefore, do the symptoms illustrate the parts affected; but the stomach is not exclusively supplied by the sympathetic filaments of the thoracic ganglia, nor is the nervous energy of the heart limited to those of the neck. These organs derive nervous power from the cerebro-spinal system by means of the pneumo-gastric nerve, and from the sympathetic system through filaments sent off from the ganglia, and the question naturally arises, may not both of these sources of nervous influence be tainted, and may not their taint conjointly produce that disordered state of the stomach, heart, and lungs which we have just described?

"The prosecution of this part of the subject will be best facilitated by investigating the following queries.

"1. Is the muscular action of the heart, arteries, stomach, and intestines, dependent upon the cerebro-spinal or upon the sympathetic system?

"2. Are painful affections of the heart, lungs, stomach and intestines, seated in the filaments of the pneumo-gastric or of the sympathetic nerves?

"3. Is the pneumo-gastric nerve the *only* nervous agent in digestion, or do the nerves of the sympathetic system exert any considerable influence in the digestive process?" 57.

The first of these questions few enlightened physiologists of the present day can find much difficulty in solving. The well-known and frequently recorded fact, that full grown fœtuses have been born without either brain, or spinal marrow, and the experiments of Le Gallois, Clift, and W. Philip, in which the whole spinal marrow and brain were destroyed without affecting in any very appreciable degree the action of the stomach and intestines, or even of the heart so long as respiration was continued, are quite conclusive as to the immediate independence, at least, of the muscular agency of the heart and stomach upon the cerebro-spinal system; and the objections which have been drawn against this view from the influence of mental emotion and from the changes wrought upon the pulse by the application of stimulants and sedatives to the brain and spinal marrow, must be regarded after the experiments above alluded to of very inferior weight.

But the two remaining queries are more difficult of solution, and very distinguished writers may be found the advocates of both sides. Desportes believed that painful affections in the heart and lungs depended on the pneumo-gastric nerve; Mr. Broughton asserted that this nerve was insensible, and Laennec imagined that the filaments of the sympathetic, as well as those of the pneumo-gastric, might be obnoxious to disease and might indiscriminately constitute the seat of pain. The portio dura of the seventh pair, the author observes, is very strikingly like the pneumo-gastric nerve in several important particulars. They arise by a single set of fibrils from a distinct part of the spinal marrow, in contradistinction to the fifth pair and the spinal nerves which communicate sensibility; in many animals they are connected towards their origin; in birds they arise together, and in fishes the substitute for the portio dura is a branch of the eighth pair. These anatomical features of resemblance create a probability that the functions of these nerves are very much alike, and as the experiments of Mr. Bell have clearly ascertained that the portio dura may be touched, stimulated and even cut without pain, Mr. Teale presumes that the pneumo-gastric nerve is equally insensible, and that, therefore, it cannot be the seat of pain. The following experiments of Mr. Broughton, upon the par vagum of a horse, abundantly confirm the justice of this presumption.

"The par vagum was exposed in the neck on one side, and insulated from its cellular connexions, but carefully retained in its place. It was repeatedly transfixed with a pin, pinched, and slowly cut through with scissors, and not the slightest degree of sensation was manifested. When pulled out from its natural position, or squeezed by the forceps, the animal appeared to wheeze as in obstructed respiration, but exhibited nothing like the twitches and startings which peculiarly mark the production of pain in irritating sensible nerves." 62.

The well known experiments of Drs. Philip and Hastings have demonstrated the importance of the eighth pair of nerves in the function of digestion; but Le Gallois, from similar experiments, concluded that digestion is not invariably suspended by their division: Magendie and others have shown that, after these nerves are cut above the cardiac orifice of the stomach, digestion still proceeded, and it is not to be denied that many animals, which are destitute of the eighth pair, can digest the coarsest nutriment with perfect ease. These circumstances prove that, although the par vagum is materially concerned in the digestive function, yet digestion can proceed without them, and since the sympathetic is the only other source of nervous supply this nerve must have some community of office with the eighth pair. Besides, it has been shown, while considering the first question, that the muscular action of the stomach is dependent upon the sympathetic, and if this muscular action become deranged in consequence of disease in this nerve, the food cannot be removed by the action of the stomach as it is digested, and dyspeptic symptoms will result as certainly as if the gastric secretions had been disordered, or deficient. It even appears probable to the author that the secretions themselves occasionally become diseased when the sympathetic is affected, because in cases attended with tenderness of the spine in the neighbourhood of the splanchnic ganglia, and in which there was no reason to suspect disease at the origin of the par vagum, he has seen large quantities of air and acid fluid secreted by the stomach; and in this view he is strongly supported by Lobstein.

"I will briefly recapitulate the inferences which appear deducible from the preceding observations.

"That painful affections of the nerves of the heart, lungs and stomach, are not seated in the filaments of the pneumo-gastric nerve, since this nerve is not a nerve of sensation, and therefore cannot be the seat of pain; consequently that they must be seated in the filaments of the sympathetic.

"That the action of the blood-vessels and muscular viscera is dependent upon the sympathetic, and consequently that irregularities in the action of these involuntary muscles may with much greater probability be referred to disease in the sympathetic than in the cerebro-spinal system.

"That as digestion has been observed to take place in some instances after the division of the eighth pair, and that it proceeds in animals which have not this nerve distributed to the stomach, it is evident that some other system of nerves (the sympathetic) exerts a considerable influence in digestion, and consequently that disease in the sympathetic may disorder or interrupt the digestive process.

"I must now refer to the pathological principle with which I commenced; namely, that disease of the nervous masses is not so much evinced by symptoms in the immediate seat of disease, as by phenomena exhibited in those remote parts to which the nerves arising from the diseased portion are distributed. Upon this principle those nervous diseases of the heart, lungs, and stomach, which have been shewn to be more probably dependent upon the sympathetic than upon the cerebro-spinal system of nerves, *should not be regarded as diseases of the particular filaments distributed to these organs, but as diseases of the ganglia or masses from which the filaments are derived.*

"The *probability* that these diseases depend upon an affection of particular ganglia is still further corroborated by the fact that tenderness may generally be detected in that part of the spine which is contiguous to the particular ganglia. Thus, when the heart is affected, there is tenderness in the cervical vertebræ; when the stomach is affected, the tenderness is seated in the middle or lower dorsal portion of the spine. The result of treatment directed to these parts may be still further adduced in corroboration." 65.

NEURALGIA OF THE HEART. It not unfrequently occurs that the action of the heart betrays signs of irregularity which cannot be ascribed to structural disease. When excited by mental emotion, or stimulated by exercise, it palpitates with unusual frequency and force; when the causes of its disturbance have ceased to operate, the irregularity continues unabated, and after variable intervals returns without any sufficiently obvious reason. Females seem to be more obnoxious to these "nervous palpitations" than males ; as the complaint advances the paroxysms become more frequent, are of longer duration, are induced by more trifling causes, and are separated by less distinct intermissions ; and ultimately to speak or move is quite sufficient to induce them. These palpitations are often accompanied by pains in the heart and lungs, not very unlike rheumatism, which are sometimes seated in the arch of the aorta, sometimes pursue the course of its large vessels, and when they attack the bronchial tubes produce strong asthmatic symptoms. This state of the heart and lungs the author believes to depend "generally, if not always" upon a morbid state of the cervical ganglia of the sympathetic ; but as the spinal cord is rarely healthy when these ganglia are diseased, it is not unusual to find that these neuralgic symptoms of the heart and lungs are complicated with darting pains along the cutaneous nerves of the head and neck, fixed pains around the shoulders, pains, numbness and tremors of the arms, and, what is somewhat singular, the left side is more frequently, as well as more violently affected than the right.

"Palpitations, purely nervous, are principally distinguished from palpitations dependent upon organic disease of the heart, by the absence of other symptoms which denote a change of structure in that organ ; in hypertrophy, the pulsations of the heart are more vehement and more uniform ; in dilatation, they are felt over an unnatural extent of the chest ; when there is obstruction to the circulation from contracted orifices, from loss of function in the valves, or from morbid alterations of the muscular structure, there are generally, in a greater or less degree, blueness, œdema, &c. These symptoms, in general, are sufficient to distinguish the two affections ; I will, however, add to them the stethoscopic distinctions enumerated by Laennec : 1. The heart is found to be of natural size ; the sound, though clear, is not strongly heard over a great extent. 2. The shock, although apparently strong at first, has, in reality, but little impulse, for it does not sensibly elevate the head of the observer. The last sign he regards as most important, when, in addition to it, we consider the frequency of the pulsations, which is always greater than natural." 45.

The two succeeding cases are good illustrations of this form of neuralgia. Mrs. H. aged 53, had been for many years subject to palpitations and dyspnœa, which were supposed to depend upon the presence of water in the chest, which varied much in intensity at different periods, and became so severe in September, 1828, that they occurred repeatedly in the day, and during night not unfrequently awoke her. The action of the heart was very violent, the dyspnœa occasionally threatened suffocation, a wheezing sound was heard in the upper part of the chest, and when the hand was applied to it a peculiar vibration could be perceived. Each paroxysm lasted about 15 minutes, and in the interval the lungs and heart resumed their natural action. She was also annoyed with "fluttering" sensations in the arms, twitchings in the muscles, stiffness of neck, and a hoarse cough, unattended with expectoration. The third, fourth, and fifth cervical vertebræ were very, and a few of the superior dorsal were somewhat tender, in consequence of which leeches were applied with immediate relief, and the next day a blister produced still greater ease. On the 15th the palpitations and twitchings became more severe, another blister was recommended, and by the 22d she felt quite well. Her complaint again returned on the 20th of December, and was again removed by leeching and blistering; she continued well until May, when another slight attack called for the employment of the same remedies, and up to the 29th of August, of the present year, she has not only remained free from every neuralgic symptom of consequence, but has gained flesh and enjoyed good health.

Sarah B. aged 17, had been five months affected with pain in the region of the heart, and palpitations which occur in paroxysms that are very violent during day, and less severe at night. The pain over the heart occasionally extended to the region of the lungs, the arms and scalp were often attacked with darting pains, an oppressive tightness was felt across the sternum, and there was great tenderness of the five superior vertebræ of the neck. By employing the ordinary remedies—leeches and blisters—and using the saline mixture for slight febrile symptoms, the palpitations and pains gradually disappeared, and in little more than a fortnight she was perfectly restored to health.

Whether, and how far these symptomatic derangements of the heart predispose to organic disease are questions of great interest; but the facts, which we yet possess upon these points, are so few, that it is impossible to enter into them with any prospect of a satisfactory result. When a muscle is forcibly and frequently exercised, an increased supply of blood and nervous energy is sent to it, that the demands made upon it in consequence of increased action may be fully satisfied. This extra supply is followed by extra growth, this extra growth gives rise to augmented energy, and in process of time both the form and function of the excited organ experience considerable change. In the brawny arms of the blacksmith, and in the muscular legs of the pedestrian, these remarks are strongly substantiated; and we are not aware of any principle in the animal economy, which prohibits our extension of this train of argument from external to internal organs. The heart is a muscle quite as much as the biceps, or gastrocnemius; and although it may not be as sensibly alive to mental stimuli as they are, it is subject like them to all the laws of muscular agency and organic life. Why therefore, may not over excitement largely and long applied modify the anatomy of this organ as well as that of any other muscle, and why may not hypertrophy, or flaccidity of its walls succeed to an increased, or diminished condition of its function, as a limb emaciates from disuse or enlarges from exercise? Laennec admits the possibility of such consequences, while he has never met with any examples, and the author confesses himself unable to speak with decision either in favour or against it; so that at present the subject lies quite open to investigation, and we hope that its great interest will procure for it the attention it requires and deserves. There is no doubt but that organic disease of the heart may exist in connexion with neuralgic symptoms dependent on either spinal, or ganglionic irritation, that they may mutually aggravate each other, and that treatment, exclusively limited to the removal of the nervous irritation, has been found materially to relieve the organic disease. Whether, therefore, it be true or otherwise, that what was mere neuralgia of the heart may ultimately issue in structural alteration, it is in every instance of heart-affection prudent, if not necessary, to attend to the condition of the spinal column.

"The treatment of nervous palpitations and neuralgic affections of the heart and lungs, has in general proved very unsatisfactory. The means employed as remedies have been various in the extreme. These complaints have been treated by anodynes, antispasmodics, and tonics; by bleeding, digitalis, and prussic acid; by electricity, galvanism, and magnetism; and by irritants and depletory measures applied to the *anterior* parts of the chest. These means have generally failed to give relief, and some of them have even aggravated the disease. Not unfrequently has it happened that the unfortunate subject of nervous palpitations, after having tried in succession almost innumerable remedies, and having repeatedly changed his medical attendant, is obliged to endure with patience his distressing nervous companions, and console himself with the assurance that his complaint is '*seldom attended with danger*.' I feel considerable confidence in stating, that when the disease is treated upon the principle which I have laid down, namely, of referring the palpitations and pains in the heart to disease of the cervical ganglia, the most beneficial results will, in the generality of cases, be obtained." 47.

Neuralgia of the Stomach. We believe it is now very generally admitted that an irregular condition of the stomach, depending on chronic inflammation of its mucous coat, is a frequent source of dyspepsia and its lengthened train of disastrous symptoms. This state may be relieved or removed by proper diet, occasional leeching and gentle sedatives. Another form of dyspepsia results from direct debility of the digestive organs, as a consequence of previous disease, and may be beneficially treated with tonic medicines. But there is a third variety of gastric ailment, which neither leeching and abstinence on the one hand, nor tonics and nutriment on the other can affect, yet the majority of its accompanying phenomena are so similar to those of the preceding cases, that they may be easily confounded. Its principal symptoms are impaired digestion, giving rise to acidity and distention; pain in the stomach, which may be confined to a small compass or be diffused over the whole epigastric region; flatulency, depending not on the decomposition of indigested aliment, but on the secretion of air by a disordered stomach, pyrosis, pulsation in the epigastre, a corded sensation around the waist, soreness along the edges of the ribs, pain in the intercostal and abdominal muscles, and other indications of disease in the spinal nerves. Indigestion and gastrodynia, the first two of the preceding symptoms, are common to gastritis and gastric neuralgia, and cannot be depended on; but the author thinks that the others are very diagnostic, more especially when attended, as they usually are, with tenderness of the spine. Flatulence from secretion, he believes to originate seldom in gastritis, pyrosis never, and a sense of constriction around the waist, soreness of the ribs and muscular pains rarely accompany it, while they are seldom absent from ganglionic disease. Præ aliis vero, says Lobstein, symptomatibus eminet flatuum extricatio, seu *pneumatosis*, e nervorum actione perversa oriunda. It must be remembered, however, that these points are not yet sufficiently well understood to admit of positive opinion, and it is certain that if disease of the ganglia continue long, the mucous membrane of the stomach, exposed as it is to continued irritation, may fall into an inflamed state, and thus may appear such an intermixture of idiopathic and sympathetic disease as shall require our treatment to be divided between the epigastre and the spine. Had the following case fallen under the care of some of our London brethren, the class of medicines which would have been adopted, it is not difficult to conjecture.

A married lady, aged 23, (June 5th, 1828) has been complaining for the last five weeks of a fixed pain in the left side of the abdomen, which is increased by pressure, an oppressive weight of the stomach after eating, constant weariness, extreme debility, a sense of constriction around the waist, which during night is distressingly severe, and an afflicting pulsation in the epigastre, which never ceases for a moment. All these symptoms are very much aggravated by taking food, and the stomach is so irritable after eating that it regurgitates by mouthfuls what has been swallowed, until it again becomes empty. Slight flatulence and acidity are constant attendants on the digestive process. On examining the spine the 7th, 8th, 9th, and 10th dorsal vertebræ betray much tenderness, and some of those both below and above are rather uneasy when pressed. Aching pains are also complained of in the legs, the skin covering the thighs is sore when rubbed, and prickling sensations are felt in the course of the saphena vein ; blister to tender vertebræ ; 10th, so much better as to say that she is "not like the same being." Pain and oppression of epigastre gone, pulsation diminished, food not rejected, is free from flatulence and acidity, lower extremities unafflicted with either prickling or pain ; but as the spine was still somewhat tender a second blister was applied with the effect of banishing every morbid feeling.

An emaciated old woman (June 11th, 1828) has complained for several months of periodical pains across the epigastre, resembling cramp, a corded sensation round the waist, sudden and copious discharges of air from the stomach, sometimes continuing for an hour at once, and pyrosis in a severe degree. The 4th, 5th, 7th, and lower dorsal vertebræ were very tender under pressure, but there was no affection of the extremities. A blister had been applied to the epigastre without relief ; blister to the lower dorsal vertebræ. (19th) Pyrosis and sense of stricture gone, flatulence much diminished, fifth dorsal vertebra still tender ; six leeches to this vertebra. On the 24th flatulence was trifling, and on the 2d of July every symptom of disease was gone.

ANGINA PECTORIS. There are few diseases of which we know so little with any certainty as that to which Heberden gave the name of *Angina Pectoris.* The symptoms, by which it is described by various authors, are more remarkable for their variety than any thing else ; the pathological products discovered after death are not much more uniform than the symptoms, and its treatment has experienced as many vicissitudes as the theories, which have been fabricated to explain its origin. Dr. Heberden observes that a disagreeable sensation in the breast comes on while walking, more especially after meals, and vanishes the moment that we cease to move. Dr. Walls describes it as a pain under the sternum, extending on each side across the breast, and affecting one or both arms where the pectoral muscle is inserted into the os humeri. Dr. Fothergill relates a case in which the leading symptoms were a sense of tightness around the chest, in a line with the mammæ, and a pungent pain under the left breast, which extended to the elbow of the left arm. Dr. Butter considers that the paroxysms are marked by dyspnœa and flatulence, and that relief is often obtained by eructation. Dyspnœa is excluded from the catalogue of symptoms by Dr. Parry, and he relates a well-marked case in which there was neither pain in the arms, or chest. Dr. Blackall considers that palpitations are frequently characteristic of this disease, while Burns maintains that palpitations are incompatible with its existence. Parry describes it as depending on an ossified state of the coronary arteries, Desportes regards it as an affection which has its seat in the pneumogastric nerve, and Laennec believes it may originate in a diseased state of any of the nerves which supply the chest and neighbouring parts. The author agrees with these last writers in maintaining that angina pectoris has its seat in the nervous system; but, in place of considering with them that the nervous filaments are the parts diseased, "dans les filets que le cœur reçoit du grand sympathétique," he only regards them as the channel of intercourse, through which some disease in the spinal cord or sympathetic ganglia draws within its influence the functions of the heart. It is certain that symptoms of angina frequently occur and are removed, proving that there can be no serious disease in the structure of this organ; and how often have the coronary arteries been found ossified, where no syncopic symptoms had been visible? Looking upon the nervous filaments themselves as the morbid agents, treatment has commenced too generally at the wrong end, and blisters, issues and such remedies have been crowded on the chest; but the author thinks it much more consistent with facts to refer all the symptoms of disease to the nervous masses from which these nerves arise, and to direct our remedial measures to the spine in preference to the chest. Tightness round the waist, oppression at the epigastre, and pains in the intercostal and abdominal muscles he traces to the lower portion of the dorsal spine; flatulence and pyrosis to the lower thoracic ganglia of the sympathetic pairs; numbness in the neck and upper extremities to the cervical division of the spinal cord; palpitations and painful affections of the heart and lungs to the cervical ganglia.

"I have been induced to refer the various groups of symptoms which have been described as angina pectoris, to an affection of some portion or portions of the spinal marrow, and of the corresponding ganglia of the sympathetic, by the following considerations.

"1. The fact, as I have before observed, that most of the morbid phenomena exhibited in the extreme filaments of nerves, are seldom owing to disease in the nerves themselves, but to an affection of the nervous mass from which they are derived.

"2. The co-existence of pain on pressing some portion of the spine with the symptoms constituting angina pectoris; and the correspondence of the painful part of the spine with the particular symptoms which are present; namely, tenderness in the lower dorsal portion of the spine in conjunction with the stomach affection, constriction, &c. and tenderness in the cervical spine, with pains in the arms, breast, and shoulders, and palpitations.

"3. The relief obtained by local antiphlogistic measures to the spine; for instance, to the lower dorsal portion when the stomach is affected, and there is constriction, and to the cervical portion when there is an affection of the arms and palpitations." 108.

Our limits allow us room for only one example and it is an important one, as showing how closely connected the ordinary symptoms of spinal irritation are with those ascribed to angina pectoris, and tending to the presumption that they not only all depend on one common cause, but that they may pass and repass into each other, as this cause is moderate or intense.

A lady, aged 56, applied to the author (on the 18th of August, 1828) in consequence of general muscular debility, palpitations, sense of epigastric tightness, and flatulency. Most of the cervical and some of the dorsal vertebræ being tender, leeches were first ordered and afterwards a blister, which gave such relief that in a few days she acknowledged herself better than she had been for many months. But on the evening of the 25th she was suddenly seized with coldness, an inexpressible sense of suffocation, tightness and oppression of chest, pain darting from the left arm into the elbow and down from the neck to the left breast, with a frightful feeling of impending death. A discharge of air from the stomach gave some relief, and a sinapism to the spine, warmth to the extremities, and internal stimuli soon restored her to a state of comparative ease. As the spine was again tender, and the symptoms at first complained of were again visible, blisters were recommended; but the patient, being obliged to return to her family, was removed from Mr. Teale's care, and the final result is unknown.

The diseases, which have now been described, appertain more especially to the heart, lungs and stomach; but the author believes that other organs are occasionally affected in a similar way and from the same cause. The small and large intestines, the kidneys, bladder and uterus are not unfrequently the seat of neuralgia depending on spinal disease, and remedial by means directed to the tender vertebræ. Hodie certissime evictum est, says Lobstein, quod tot numerosæ sensationes quæ in epigastrio percipiuntur, neque ad musculos, neque ad vasa, neque ad organa gastrica sint referenda; sed magis ad plexum nervosum gangliosum trunco cœliaco insidentem. Autenreith likewise observes (in the first volume of the Jübinger Blœtter für naturwissenschaft und Arzneykunde) that in the bodies of those who have died of typhus, he has occasionally seen the abdominal nerves altered in appearance.

"It is of great importance to bear in mind the circumstance that these nervous affections sometimes accompany other diseases. When the vertebræ, or intervertebral cartilages are inflamed, the neighbouring nervous tissues often participate, and neuralgic symptoms are the result. These nervous affections often constitute the most distressing part of the complaint, and, by proper attention to them the sufferings of the patient may from time to time be alleviated during the lingering progress of the vertebral disease. In fevers, symptoms of a neuralgic character often make their appearance, and aggravate the sufferings of the patient. The following case lately occurred to me. A young lady, having proceeded in a favourable manner for two or three weeks under common fever, became affected in the afternoon with paroxysms of oppression in respiration, attended with severe aching pain and constriction round the waist. These symptoms returned about the same hour for four or five days, gradually increasing in violence until they became truly alarming; tenderness was discovered in two or three of the dorsal vertebræ, and a few leeches applied to the painful part, prevented the recurrence of the attacks. The fever afterwards pursued the usual course, and ultimately terminated favourably. Neuralgic affections of the scalp, connected with tenderness in the cervical vertebræ, often occur in fever, and are sometimes mistaken for pain of the encephalon. In phthisis, pains in the intercostal muscles, and oppression of respiration, are often of a neuralgic character, and readily admit of alleviation; the more formidable disease of the lungs, however, seems to predispose to their recurrence.

"Dr. Brown has observed neuralgic pains in the neck and scalp accompanying severe inflammatory affections of the fauces, and has also met with similar symptoms in conjunction with hepatitis. My own observation enables me to confirm these remarks of Dr. Brown. The principal neuralgic symptoms which I have observed in conjunction with hepatitis, are constriction across the epigastrium and pain or tenderness along the cartilages of the ribs. This pain is sometimes supposed to be seated in the liver when the right side is affected, but a precisely similar affection is as frequently met with on the left. I have known this neuralgic affection to be treated as hepatitis when there has not been any real evidence of disease of the liver. A patient is now under my care, who is suffering from hepatitis, as denoted by yellowness of the skin, bilious urine, clay-coloured fæces, and deep-seated tenderness beneath the cartilages of the ribs; during the course of this complaint, he was for several mornings in succession attacked, about five o'clock, with pain and constriction across the epigastrium which he compared to cramp, flatulent distention of the stomach and intestines, pain along the cartilages of the lower ribs on each side, and on pressing these parts a degree of soreness was felt; the attacks continued from one to two hours, during which great restlessness was produced. Tenderness was detected in the vertebræ, and a blister has quite removed the paroxysms.

"These circumstances point out the important fact that irritation of the capillary expansions of nerves may sometimes excite actual disease in the parts where the nerves originate." 55.

With a few remarks upon the probable connexion between colica pictonum and nervous disease, and a cautionary admonition to the reader against supposing that he has been either advocating an impregnable theory, or a practice which must ever prove infallible, Mr. Teale concludes his little work, and it were quite superfluous in us to repeat in plainer language than has been already used, the estimate which we have formed of its execution. Suffice it to remark that, although we do not look upon his plan of cure in neuralgic disease as proof against disappointment, we consider it one of the most promising which has ever been recommended; and, although we must as yet hold his views of this disease as theoretical, until dissection supply the *experimentum crucis*, we are troubled with as few misgivings on the subject of its orthodoxy, as any can be during the absence of the *chief witness*. Mr. Teale has done much in a little book to elucidate an obscure order of diseases, and we have no doubt but that his labors will receive that recompense which talent and industry deserve.

---

